# Clinical, Virological, and Pathological Profile of Patients Who Died of COVID-19: An Autopsy-Based Study From India

**DOI:** 10.7759/cureus.23538

**Published:** 2022-03-27

**Authors:** Jayanthi Yadav, Garima Goel, Shashank Purwar, Saurabh Saigal, Ashwani Tandon, Ankur Joshi, Brinda Patel, Sravan JS, Mahaluxmi S, Jitendra Singh, Prem Shankar, Arneet Arora, Sarman Singh

**Affiliations:** 1 Forensic Medicine, All India Institute of Medical Sciences, Bhopal, Bhopal, IND; 2 Pathology, All India Institute of Medical Sciences, Bhopal, Bhopal, IND; 3 Microbiology, All India Institute of Medical Sciences, Bhopal, Bhopal, IND; 4 Anaesthesiology, All India Institute of Medical Sciences, Bhopal, Bhopal, IND; 5 Pathology and Laboratory Medicine, All India Institute of Medical Sciences, Bhopal, Bhopal, IND; 6 Community and Family Medicine, All India Institute of Medical Sciences, Bhopal, Bhopal, IND; 7 Forensic Medicine, Netaji Subhash Chandra Bose Medical College, Jabalpur, IND; 8 Translation Medicine, All India Institute of Medical Sciences, Bhopal, Bhopal, IND

**Keywords:** covid autopsy of liver and kidney, covid autopsy of lung, covid 19 india, histopathology of lungs in covid 19, virology of tissues in covid 19, covid 19 clinioicopathologic features, covid 19 autopsy

## Abstract

Background and objective

Ever since its emergence in December 2019, coronavirus disease 2019 (COVID-19) has affected more than 220 million people worldwide, resulting in more than 45 million deaths. The present autopsy-based study was undertaken to understand the pathophysiology of the disease and correlate the histopathological and virological findings with the antemortem clinical and biochemical determinants.

Methods

In this prospective observational study, autopsies were carried out on 21 reverse transcription-polymerase chain reaction (RT-PCR)-proven COVID-19 patients who had died of the disease. The histopathological findings of tissue samples from lungs, liver, and kidneys collected during the autopsy were graded based on their presence or absence; if present, they were graded as either focal or diffuse. The findings were correlated with antemortem clinical and biochemical findings. Postmortem tissue RT-PCR analysis was conducted, and findings were compared with postmortem histopathological findings.

Results

There was multisystem involvement with the COVID-19 cases. The involvement of lungs was observed in most of the cases (90.4%). The presence of viral RNA was observed in all the organs including the liver (57.1%) and kidney (66.6%). An association was observed between antemortem biochemical parameters [aspartate aminotransferase (AST), alanine aminotransferase (ALT)] and the histopathological features in the liver. No correlation between the Sequential Organ Failure Assessment (SOFA) score recorded clinically and lung histopathology was observed; nor was there any correlation between blood urea-creatinine levels and kidney histopathology.

Conclusions

Our study shows that COVID-19 is a multisystemic disease and the mortality associated with it is likely to be multifactorial. Despite the presence of amplifiable severe acute respiratory syndrome coronavirus 2 (SARS-CoV-2) in various organs, no association could be established between the clinical and histopathology findings. Neither the duration of hospitalization nor the duration of mechanical ventilation showed any correlation with the severity of histopathological findings in the lungs at autopsy.

## Introduction

Coronavirus disease 2019 (COVID-19) is caused by a single-stranded RNA virus known as severe acute respiratory syndrome coronavirus 2 (SARS-CoV-2), which has spread to the entire world since it was first identified in December 2019 in Wuhan in the Hubei province of China [1]. The first confirmed case of COVID-19 in India was reported on January 30, 2020, and since then, more than 0.3 billion people have been infected and more than 500,000 people have died in India alone.

The clinical presentation and course of the disease vary considerably from one individual to another [2]. Several studies to determine the pathophysiology of the disease have found that SARS-CoV-2 adheres to angiotensin-converting enzyme 2 (ACE2) receptors, which are almost ubiquitous on several types of human cells [3,4]. However, the exact process leading to multiple organ dysfunction and fatal complications in COVID-19 has not been described adequately and the role of coexisting morbidities leading to mortality has not been elucidated. Autopsy findings are fundamental for a better understanding of the pathophysiology of a disease. Autopsy studies on COVID-19 have been published describing the common histopathological findings, but the majority of these studies are based on the European or Western population and have only documented the pathological findings in various organs individually in COVID-19 patients [1,5-9]. To the best of our knowledge, no complete autopsy study has been conducted in India previously. In the present study, complete clinical autopsies were carried out at a tertiary care center in Central India followed by histopathological and qualitative reverse transcription-polymerase chain reaction (RT-PCR) tests of the tissue samples, and an attempt was made to correlate the postmortem findings with antemortem clinical findings. The histopathological examination of the organs was performed to evaluate the presence of any features specific to the viral injury and to understand the mechanism of injury to the organ. RT-PCR was done at the autopsy to determine the presence of viral RNA in various organs. An attempt was made to correlate the histopathological findings with the antemortem clinical findings such as the duration of hospitalization and mechanical ventilation, comorbidities, biochemical parameters, and the results of RT-PCR of the tissues.

## Materials and methods

This prospective study was carried out between August and October 2020, during the first wave of the COVID-19 pandemic in India. During this period, 148 patients with RT-PCR-confirmed COVID-19 died at our center. Our center is located in Central India and is a government-run tertiary care center with a well-developed ICU and other facilities with a bed strength of more than 800. At that time, our center was converted to a dedicated COVID-19 care center, and only patients having COVID-19 were admitted. The next of kin of 134 deceased patients were approached for consent for autopsy. However, consent could only be obtained for 21 cases, in which complete autopsies were performed. The demographic and clinical details were retrieved from the case records of the deceased patients. The study was approved by the Institutional Ethics Committee of our institution (vide letter no. IHEC-LOP/2020/IM0273 dated 31/7/2020).

Autopsy procedure

The autopsy was carried out in accordance with the available guidelines and standard operating protocols (CDC, RCP, Government of India/ICMR, and WHO) in the BSL 2 autopsy room with heating, ventilation, and air conditioning (HVAC) facilities. Efforts were made to obtain consent for autopsy and conduct the autopsy within a short period after death so that postmortem interval (PMI) remained short and autolytic changes could be minimized. Low aerosol-generating techniques were adopted during autopsy to limit the spread of infection [10]. The specimens and swabs from different organs for histopathological and RT-PCR analysis were collected and transported to the specific laboratories within the institute in recommended fixatives following the biosafety measures (special care was taken to avoid cross-contamination of samples during the autopsy procedures as per CDC and RCP guidelines).

SARS-CoV-2 RT-PCR

The qualitative RT-PCR was performed on autopsy samples of nasopharyngeal and tracheal swabs and on the tissue samples from both lungs, liver, and kidneys. The RNA isolation was done using a viral DNA/RNA extraction kit manufactured by Wuhan MGI Tech Co. Ltd, Wuhan, China, as per the manufacturer’s instructions. The extracted RNA samples were subjected to RT-PCR using ICMR-NIV Pune, Allplex^TM^ 2019-nCOV assay, LabGun^TM^ COVID-19 RT-PCR Kit, TaqPath^TM ^COVID-19 combo kit, BGI Real-Time Fluorescent RT-PCR Kit, and TRUPCR® SARS-CoV-2 RT qPCR KIT assays for detecting SARS-CoV-2 as per manufacturers' protocols on an ABI7500 Fast Real-Time PCR system (Applied Biosystems, Waltham, MA).

Histopathological examination

The autopsy specimens from both lungs, the liver, and kidneys for histopathology were received in 10% formalin and kept for fixation in sealed containers for at least 48 hours before grossing. The representative sections from the respective organs were processed and stained with hematoxylin and eosin stain as per the standard operating procedure of our institute. All the organs were examined for any specific virus-induced histological features mentioned in the literature by two pathologists independently to ensure that there was no observer bias. The extent of histopathological findings in the lungs, liver, and kidneys was correlated with the antemortem battery of investigations done during the hospitalization period, including D-dimer, C-reactive protein (CRP), aspartate aminotransferase (AST), alanine aminotransferase (ALT), serum urea, and creatinine. These findings were also correlated with the clinical findings related to the patients, including the duration of hospital stay and mechanical ventilation, comorbidities, and treatment history. Sequential Organ Failure Assessment (SOFA) score was calculated to assess the clinical severity of patients and it was correlated with the severity of their histopathological changes in the lungs at autopsy. The histopathological findings in organs were also correlated with the treatment outcomes, which were instituted as per national guidelines at that time [11]. While assessing the clinicopathological severity of kidneys, acute kidney injury (AKI) was classified into stages 1, 2, and 3 according to the Kidney Disease Improving Global Outcomes (KDIGO) definition for AKI [12]. The grading for acute tubular necrosis (ATN) was done as per the criteria described by Santoriello et al. [13] on the basis of distribution (mild: <50%; moderate or severe: >50%). The histopathological and biochemical findings were also correlated with the RT-PCR status of the tissue.

Statistical analysis

Data were initially checked for missing values, redundancies, outliers, and discrepancies, and then imported to R (R Core Team 2019). The data were converted to tidy format and descriptively analyzed in terms of frequency, median, and interquartile range using the {gtsummary} package. The data were visualized by creating bubble diagrams and box plots using ggplot2. As the distribution of data was non-parametric in nature, Kruskal-Wallis and Wilcoxon tests were applied to ascertain the statistically significant difference of biochemical/clinical parameter values among the histopathological extent (absent/focal/diffuse) of organ involvement. A p-value <0.5 was considered statistically significant.

## Results

A complete autopsy was performed on 21 cases including 15 males and six females who had succumbed to COVID-19 infection. All the patients had been admitted for the treatment of COVID-19. The age of the patients ranged from 25-84 years (with 58 years as the first quartile, 26 years as the median, and 72 years as the third quartile). Of these patients, 20 (95.2%) had presented with comorbidities at the time of hospital admission. Five cases (23.8%) had presented with single comorbidity whereas 15 cases (71.4%) had presented with multiple comorbidities, as listed in Table 1.

**Table 1:** Comorbidities in deceased patients at the time of admission with COVID-19 COVID-19: coronavirus disease 2019

Comorbidities	Number of cases (%)
Diabetes mellitus	15 (71.4)
Hypertension	13 (61.9)
Obesity	6 (28.5)
Hypothyroidism	4 (19)
Cardiac disease	3 (14.2)
Cerebrovascular disease	1 (4.7)
Hepatitis B	1 (4.7)
Multiple myeloma	1 (4.7)
Pancreatitis	1 (4.7)
Chronic obstructive pulmonary disease	1 (4.7)
Chronic Kidney disease	1 (4.7)

The autopsy was carried out as soon as possible after receiving consent from the family to avoid autolytic changes in the tissue. The time from death to autopsy ranged from 3.5 to 19.5 hours (12.5 hours as the median, and eight and 15 hours as the first and third quartile respectively). RT-PCR done on nasopharyngeal swab collected during the autopsy was positive in all but two cases. RT-PCR of tracheal swab collected during the autopsy was positive in all but three cases. The RT-PCR findings of various tissues and swabs collected during the autopsy and processed in our institute are depicted in Table 2. 

**Table 2:** RT-PCR status of various organs tissue and swab collected during autopsy RT-PCR: reverse transcription-polymerase chain reaction; +: positive; -: negative; NA: not available (as tissue was not collected during autopsy for RTPCR); R: rejected (as the amount of retrieved RNA was insufficient)

	Right lung tissue	Left lung tissue	Liver tissue	Kidney tissue	Nasopharyngeal swab	Tracheal swab
1	NA	NA	NA	NA	+	+
2	+	+	+	+	+	+
3	+	+	-	+	+	+
4	+	+	+	+	+	+
5	+	+	-	-	+	-
6	+	+	-	+	+	+
7	+	+	+	+	+	+
8	-	-	-	+	+	-
9	+	+	-	R	-	-
10	+	+	+	+	+	+
11	+	+	+	+	+	+
12	+	+	+	+	+	+
13	-	-	+	+	+	+
14	-	-	+	+	+	+
15	+	+	+	+	+	+
16	+	+	-	-	+	+
17	+	+	+	+	+	+
18	+	-	-	-	+	+
19	+	+	+	-	-	+
20	-	-	-	+	-	+
21	+	+	-	+	+	+

The overall hospital stay ranged from a few hours to 39 days (seven days as the median, and four days and 21 days as first and third quartile respectively). Of note, 17 patients (80.9%) were put on mechanical ventilation for 1-14 days (median of four days with one and 5.5 days as first and third quartile respectively). The treatment protocol for the patients included a combination of anticoagulants (85.7%), antibiotics (85.7%), steroids (80.9%), and antiviral drugs (33.3%) as per the national Revised Guidelines on Clinical Management of COVID-19 by the Government of India Ministry of Health & Family Welfare Directorate General of Health Services. The details of clinical findings and treatment are depicted in Table 3. Case no. 10 had been a referred case who had died on the way and brought dead to our center, and hence no treatment could be given. Case no. 20 had succumbed to the injuries after jumping from the ward where she had been admitted. 

**Table 3:** Clinical characteristics of deceased patients with COVID-19 COVID-19: coronavirus disease 2019; M: male; F: female; ARDS: acute respiratory distress syndrome; BA: bronchial asthma; CVA: cerebrovascular attack; CKD: chronic kidney disease; CAD: coronary artery disease; DM: diabetes mellitus; HTN: hypertension; HThy: hypothyroidism; MM: multiple myeloma; MODS: multiorgan dysfunction syndrome; Ob: obesity; SARI: severe acute respiratory infection; A: antibiotics; E: enoxaparin; S: steroid; R: remdesivir

Case #	Age in years/sex	Comorbidities	Drugs used for treatment (C/S/R/A)	Hospitalization (days)	Duration of mechanical ventilation (days)	Cause of death	Death-to-autopsy interval (hours)
1	58/M	HTN/Ob	E/S/R/A	16	1	Septic shock with COVID-19 pneumonia	16.5
2	67/M	DM/HTN/CAD	E/A	6	0	Cardiopulmonary shock with COVID-19 pneumonia	12.5
3	25/M	None	E/S/R/A	14	14	MODs with septic shock, ARDS with COVID-19 pneumonia	8
4	30/M	Alcoholic pancreatitis	A	2	2	MODS with alcohol-induced pancreatitis and COVID-19 pneumonia	19.5
5	60/M	DM/HTN/Ob	E/S/A	39	4	Septic shock with COVID-19 pneumonia	12
6	30/M	Type-1 DM/Hep B	A	4	0	Type I DM with recurrent hypoglycemia with UTI with sepsis	8.5
7	51/M	DM/HTN/CKD/HThy	E/S/R/A	3	4	MODS with septic shock	11
8	75/M	DM/HTN/HThy/Ob	E/S/A	6	4	MODS with sepsis and COVID-19 pneumonia	17
9	64/M	HTN	E/S/A	9	1	MODS with septic shock, ARDS, and COVID-19 pneumonia	12
10	71/M	DM/CAD/MM	-	0	0	Sudden cardiac arrest, SARI with severe anemia and multiple myeloma	5
11	62/M	DM/HTN/BA/Ob	E/S	5	5	Sepsis with MODS with ARDS and COVID-19 pneumonia	14
12	79/M	DM/Ob	E/S	8	1	COVID-19 pneumonia with ARDS and septic shock	18
13	45/F	DM/HTN/Ob	E/S/A	2	2	COVID-19 pneumonia	14.5
14	75/M	DM/HTN	E/S/R/A	8	1	Septic shock with metabolic acidosis, COVID-19 pneumonia	5
15	70/F	DM//HThy/Ob	E/S/A	10	6	Septic shock with MODS and COVID-19 pneumonia	18
16	84/M	DM/HTN/CVA	E/S/R/A	10	4	Sepsis with MODS and COVID-19 pneumonia	14.5
17	75/F	DM/HTN	E/S/A	9	1	Septic shock with COVID-19 pneumonia	15
18	72/M	DM/HTN/Ob	E/S/R/A	20	14	Cardiac arrest with COVID-19 pneumonia	3.5
19	64/F	DM//HThy/Ob	E/S/R/A	18	6	MODS with COVID-19 pneumonia	6
20	60/F	HTN/Ob	E/S/A	3	0	Multiple injuries	15
21	60/F	CAD/Ob	E/S/A	4	4	Sepsis with MODS and COVID-19 pneumonia	6

D-dimer levels were estimated in 11 out of the 21 cases at the time of ICU admission and were found to be elevated in 10 cases (90.9%). None of these patients showed the presence of macroscopic thrombi in any of the organs, whereas microthrombi in small vessels were noticed in multiple organs. The biochemical findings of all the cases are listed in Table 4.

**Table 4:** Clinico-biochemical parameters of deceased patients with COVID-19 COVID-19: coronavirus disease 2019; SOFA: Sequential Organ Failure Assessment; CRP: C-reactive protein; AST: aspartate aminotransferase; ALT: alanine aminotransferase

Case no.	SOFA score	Inflammatory markers		Liver function tests			Renal function tests	
		CRP (<5 mg/l)	D-dimer of 0.1-0.5 (microgm/mL)	Bilirubin (initial/peak) of 0.3-1.2 mg/dL	AST (initial/peak) of <50 u/L	ALT (initial/peak) of <50 u/L	Creatinine (initial/peak) of 0.6-1.2 mg/dL	Urea (initial/peak) of 20-40 mg/dL
1	3	316	0.36	1.2/1.2	22.82/148.17	25.99/130.4	1.4/1.6	32.1/78
2	8	7.63	1.06	0.4/0.5	37.6/37.6	27.5/40.5	1.4/1.8	44/75.3
3	10	285	0.69	0.72/1.8	47.8/393.8	42.9/91.4	1/3.1	26.4/216.8
4	14	590.36	-	12.5/15.8	760.4/760.4	183.9/183.9	5.4/5.6	199.1/254.3
5	8	192.07	1.77	0.99/1.25	41.27/378.6	76.9/108.3	1.2/1.2	43.9/79.1
6	NA	78.64	-	0.4/0.4	19.8/19.8	19.4/22.2	1.2/1.2	40.7/44.1
7	8	65.53	-	0.85/0.99	66.8/67.0	23.3/23.3	4/5.5	142.8/205.5
8	8	195.8	2.67	0.6/1.0	30/61.0	23.2/69.6	1.3/1.3	38.6/101.5
9	4	310	4.38	1.6/1.8	209.7/209.7	229.2/229.2	1.2/1.2	30.6/47.1
10	NA	-	-	-	-	-	-	-
11	6	323.11	2.09	0.8/0.8	48.5/67.8	23.9/33.4	1/4.2	41.4/193
12	10	169.46	-	0.5/0.6	46.9/47.3	30.6/41.8	0.9/1.3	58/119.2
13	6	96.07	-	3.18/3.45	162.1/379.6	66.9/70.3	1.7/2.2	44.2/70.1
14	2	338.37	14.21	0.5/0.9	60.1/60.1	54.3/54.3	0.9/0.9	33.7/69.4
15	6	204.68	1.84	0.4/0.5	77.5/106.6	48.4/75.1	1/2.3	34.3/189.2
16	4	25.41	-	0.3/1.6	40.9/127.1	21.7/56.5	1.1/1.1	62.6/97
17	7	149.23	-	0.5/1.2	88.2/88.2	35.3/39.7	1.4/1.4	67/201
18	4	307.3	0.73	0.6/0.97	30.31/118.4	26.5/109.6	2.6/3.7	40.4/333
19	4	96.53	0.57	0.6/1.1	48.3/104	21.1/49	0.7/1.02	24.4/73.6
20	2	-	-	0.3/0.6	50.2/50.2	49/49	0.7/0.8	-
21	NA	-	-	1.1/1.1	18.9/27.7	13.2/21.8	1.6/1.6	-

**Figure 1:**
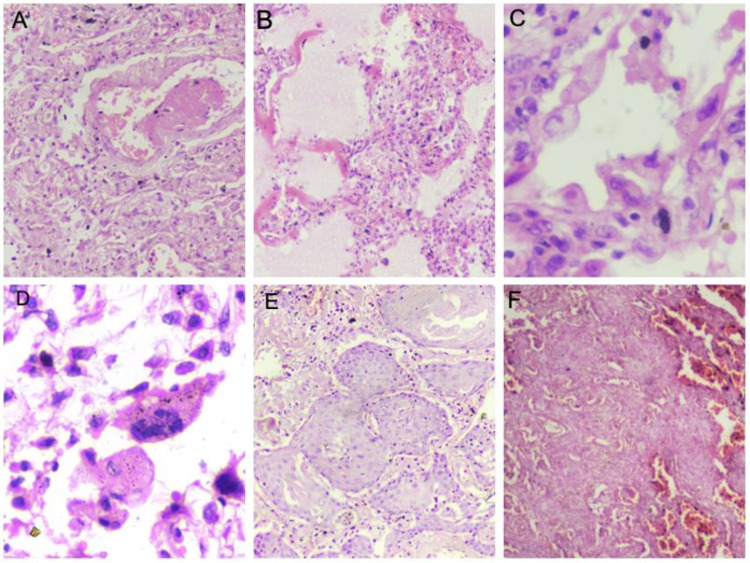
Haematoxylin and eosin-stained sections of lung parenchyma displaying various histopathological features (A) Blood vessel showing fibrin thrombi (100x). (B) Presence of hyaline membranes in exudative phase (100x). (C) Pneumocyte hyperplasia with viral cytopathic effects in proliferative phase (400x). (D) Multinucleated giant cells (400x). (E) Squamous metaplasia (100x). (F) Fibrosis of the lung parenchyma in fibrotic phase (40x)

The histopathological features of bilateral lungs from 21 autopsies are presented in Table 5 and Figure 1.

**Table 5:** Histopathological findings in lungs of deceased patients with COVID-19 COVID-19: coronavirus disease 2019

	Histopathological findings in lungs	Absent (0)	Focal (1-25%)	Diffuse (>26%)
Exudative phase	Capillary congestion	0/21	7/21	14/21
	Interstitial and Intra-alveolar edema	1/21	11/21	9/21
	Alveolar hemorrhage	11/21	6/21	4/21
	Hyaline membrane	8/21	7/21	6/21
	Dilated alveolar duct/collapsed alveoli	3/21	11/21	7/21
	Fibrin thrombi	2/21	13/21	6/21
Proliferative phase	Type 2 pneumocyte hyperplasia	3/21	5/21	13/21
	Viral cytopathic effects	3/21	5/21	13/21
	Alveolar granulation tissue	11/21	7/21	3/21
	Multinucleate giant cells	11/21	9/21	1/21
Fibrotic phase	Squamous metaplasia	14/21	5/21	2/21
	Fibroblastic proliferation	4/21	11/21	6/21
	Capillary proliferation	8/21	10/21	3/21
	Pleural involvement	16/21	5/21	0/21
	Alveolar/interstitial inflammatory infiltrate	1/21	8/21	12/21

**Figure 2:**
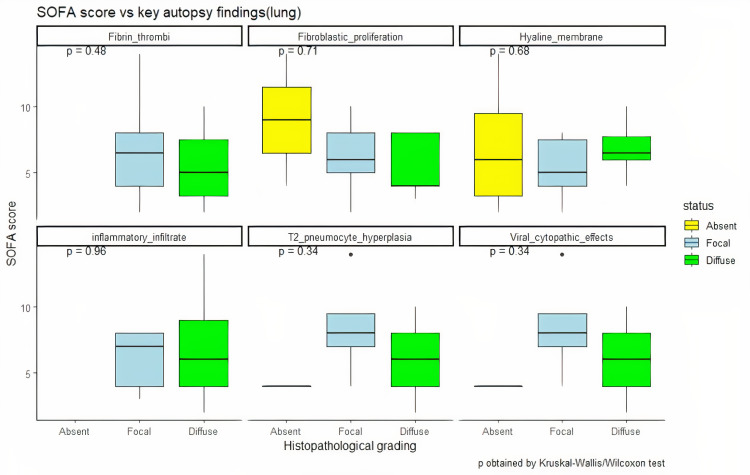
Distribution of clinical severity (SOFA score) with histopathological findings in lungs SOFA: Sequential Organ Failure Assessment

The majority (11 out of 21 cases, 52.3%) of the patients demonstrated histopathological features of the exudative and proliferative phase of diffuse alveolar damage (DAD). Eight cases (38%) showed histological features of the fibrotic phase of DAD with pleural involvement and organizing pneumonia. Two patients (9.7%) did not show any features of DAD (cases 6 and 10); of these two, one patient (case 10) had been brought dead to the emergency and hence not hospitalized. On comparison of the duration of hospitalization, mechanical ventilation, and SOFA score with histopathological features, it was observed that neither of them had any correlation with the phase of DAD (Figure 2).

Patients with hospitalization of as low as 2.5 days and no mechanical ventilation also showed features of the fibrotic phase of DAD, whereas patients with 39 days of hospitalization and 14 days of mechanical ventilation displayed overlapping features of the exudative and proliferative phase of DAD. The RT-PCR of the lung tissue was carried out in 20 cases and was positive in both the lungs in 15 cases (75%). Out of the five cases (25%) with negative RT-PCR, three cases demonstrated the fibrotic phase of DAD. The results of RT-PCR of the lung tissue had no correlation with the severity and extent of histopathological features. Seven out of the total 21 cases (33.3%) had a peak CRP of more than 250 mg/L, of which five cases displayed the fibrotic phase of DAD on histopathology.

**Figure 3:**
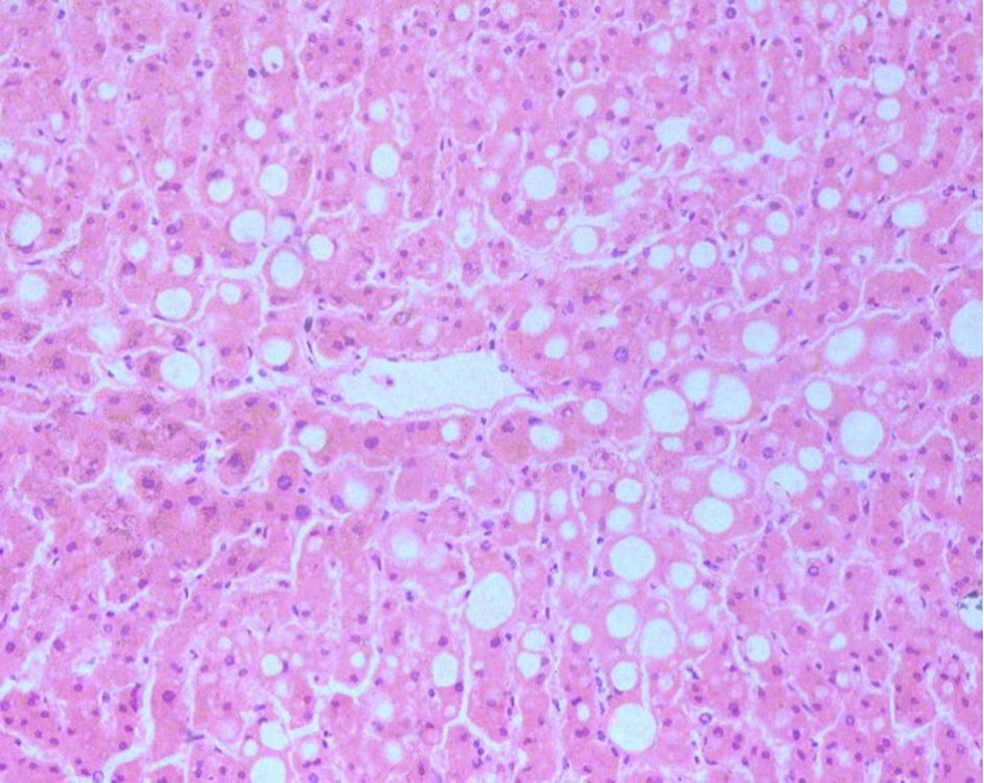
Haematoxylin and eosin-stained section of liver parenchyma displaying macrovesicular change (100x)

The histopathological changes observed in the liver are summarized in Table 6. The most common findings were periportal inflammation by lymphocytes (95.23%, n=20) and steatosis (81%, n=17) (Figure 3).

**Table 6:** The histopathological changes observed in the liver of deceased patients with COVID-19 (n=21) COVID-19: coronavirus disease 2019

Histopathological finding in the liver (n=21)	Absent (0)	Focal (1-25%)	Diffuse (>26%)
Portal inflammation	1	14	6
Steatosis	4	7	10
Portal vein dilation	5	14	2
Herniated portal vein in periportal parenchyma	12	8	1
Lobular inflammation	12	6	3
Periportal abnormal vessels	13	8	0
Portal vein fibrosis	15	6	0
Fibrosis	19	0	2
Vascular thrombosis	20	1	0
Parenchymal necrosis	20	0	1

**Figure 4:**
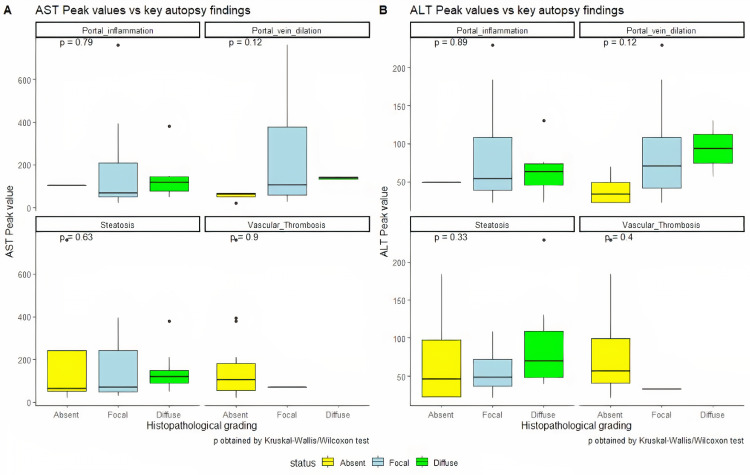
Distribution of AST and ALT values with the histopathological findings in the liver AST: aspartate aminotransferase; ALT: alanine aminotransferase

A correlation was observed between clinical AST and ALT values and autopsy histopathological findings of steatosis in the liver. The cases with normal initial and peak AST and ALT values had absent or mild steatosis (n=11) in comparison to cases with high initial and peak AST and ALT values, which presented with moderate or advanced steatosis (n=10) (Figure 4).

Patients with severe steatosis also had higher serum triglyceride levels as compared to patients with absent or mild steatosis. RT-PCR of liver tissue was carried out in 20 cases and was found to be positive for viral RNA in 11 cases (55%). The median initial AST level was higher in individuals with RT-PCR positivity in the liver tissue as compared to those with RT-PCR negativity in the liver tissue, while median peak AST, initial ALT, and peak ALT values were higher in those with RT-PCR-negative liver tissue as compared to persons with RT-PCR-positive liver tissue, as shown in Table 7.

**Table 7:** Comparison of median ALT and AST values with RT-PCR status in the liver tissue AST: aspartate aminotransferase; ALT: alanine aminotransferase; RT-PCR: reverse transcription-polymerase chain reaction

Liver enzymes	RT-PCR-positive	RT-PCR-negative
Median initial AST (IU)	60.1	41.27
Median peak AST (IU)	67.75	127.06
Median initial ALT (IU)	30.63	42.82
Median peak ALT (IU)	48.95	91.39

**Figure 5:**
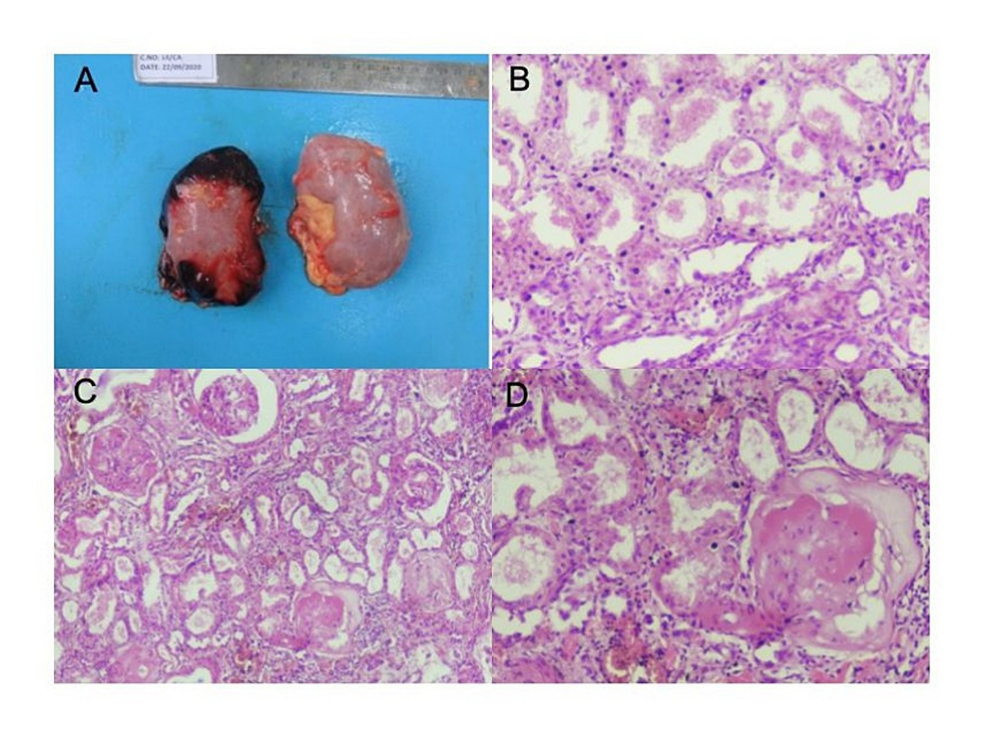
Histopathological findings of renal parenchyma (A) Gross appearance of bilateral kidney in a single case showing congestion of the external surface. (B) Acute tubular necrosis (100x). (C) Acute tubular necrosis with sclerosed glomeruli (40x). (D) Sclerosed glomerulus in diabetic nephropathy (100x)

Histopathological changes observed in kidneys of 21 COVID-19 deceased patients are presented in Table 8 and Figure 5.

**Table 8:** Histopathological changes observed in kidneys of deceased patients with COVID-19 COVID-19: coronavirus disease 2019

Histopathological changes in kidneys				
Tubules	Absent (0)	Focal (<50%)	Diffuse or severe (>50%)	Obscured by autolysis
Acute tubular necrosis	2/21	4/21	12/21	3/21
Glomeruli	Absent	25% glomeruli	26-50% glomeruli	>50% glomeruli
Glomerulosclerosis	10/21	11/21	0/21	0/21
Blood vessels and interstitium	Absent	Focal	Moderate	Diffuse
Fibrin thrombi in vessels	18/21	3/21	0/21	0/21
Arteriosclerosis	10/21	11/21	0/21	0/21
Interstitial inflammation	6/21	15/21	0/21	0/21

**Figure 6:**
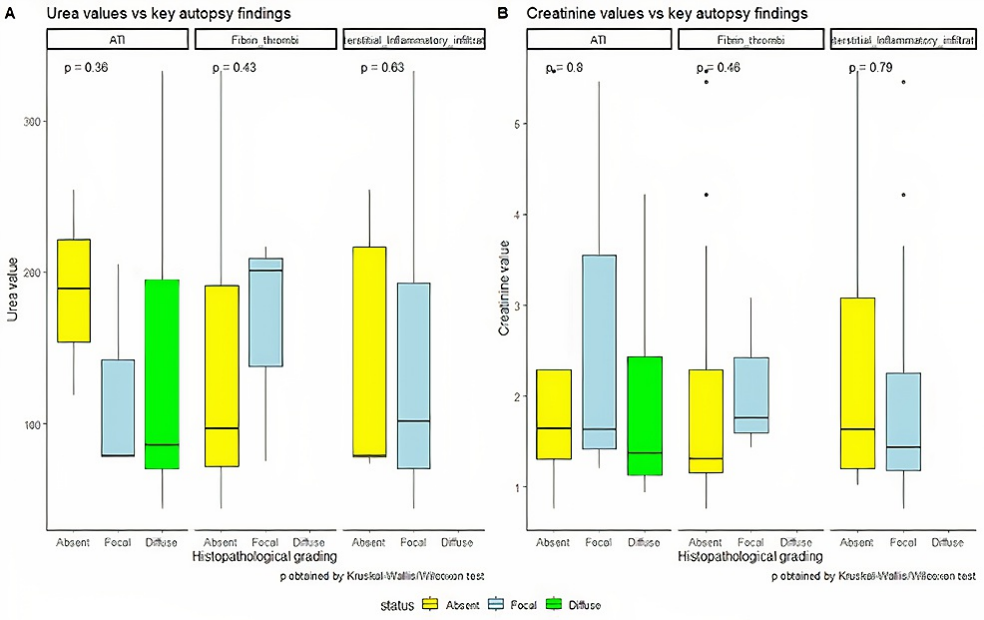
Distribution of urea and creatinine with the histopathological findings in the liver

Clinically, AKI was observed in 11 out of 21 patients (52%). Out of these eleven patients, four were in stage 1, five in stage 2, and two in stage 3 AKI. Among the 11 patients who developed AKI, five patients (45.4%) had both diabetes and hypertension, whereas three patients (27.2%) had either diabetes or hypertension, and three (27.2%) patients had no comorbidities. Interestingly, a patient with known chronic kidney disease developed stage 1 AKI during hospitalization and showed features of diabetic glomerulopathy on histopathology along with the other findings. The histopathological findings at autopsy did not show any correlation with antemortem serum urea or creatinine values recorded during hospitalization (Figure 6).

**Figure 7:**
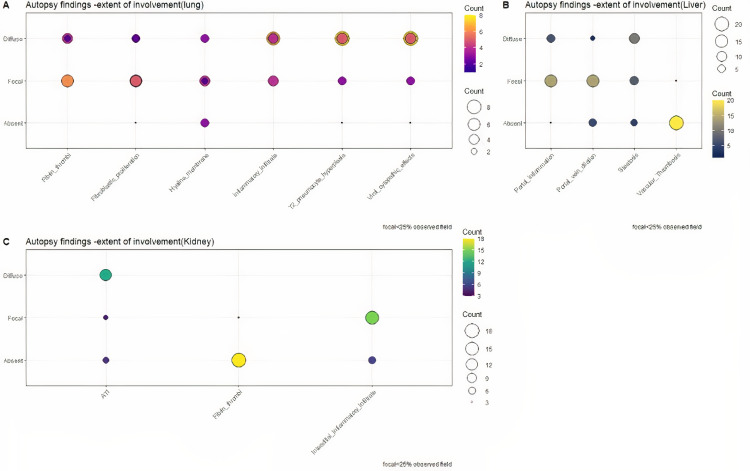
Extent of the involvement of organs (A) Extent of the involvement of lungs. (B) Extent of the involvement of the liver. (C) Extent of the involvement of kidneys

ATN was observed histopathologically after autopsy in 16 cases (76.1%). On clinical correlation, among the 12 of the 16 cases showing moderate or severe ATN, eight cases had both diabetes and hypertension. Antemortem clinical D-dimer levels were available in eight of the 12 cases with moderate or severe ATN, and D-dimer levels were elevated in all eight cases. Glomerulosclerosis was seen in 11 (52.3%) of the 21 cases with <50% involvement of glomeruli, considered as focal glomerulosclerosis. All 11 patients had a history of diabetes, while seven of them had both diabetes and hypertension. Arteriosclerosis was noted on autopsy histopathology in 11 out of 21 cases, of which 10 cases had a history of hypertension. Fibrin thrombi in vessels of kidneys were seen at autopsy in three of the 21 cases (14.3%). The RT-PCR was positive in the renal tissue in 14 cases (66.6%) whereas it was negative in seven cases (33.4%). Seven of the 14 cases with positive RT-PCR in renal tissue showed moderate or severe ATN and two cases showed mild ATN. Lungs were the main organ involved although the effect of comorbidities was seen mainly in the kidneys (Figure 7).

## Discussion

A complete autopsy plays a crucial role in determining the course and extent of the disease by allowing for sufficient tissue sampling in comparison to partial or minimally invasive autopsies. There have only been a few complete autopsy-based studies on COVID 19 [14], and to the best of our knowledge, this study is the first complete autopsy study on COVID-19 deaths from India comparing the histopathological changes in organs with clinical and laboratory parameters. There are several challenges related to infrastructure and availability of skilled human resources for conducting an autopsy and ancillary investigations in a prescribed way, as well as the availability of funds for the RT-PCR tests amidst the pandemic, histopathological sample processing, and the feasibility of study of clinical documents; however, the main impediment is to obtain consent from the next of kin for a complete autopsy. In a few countries like Germany, a complete autopsy for COVID-19 cases is mandatory by law, and hence complete autopsies can be carried out without the limiting factor of consent [7]. In the present study, complete pathological autopsies were conducted on 21 COVID-19 patients whose diagnoses had been confirmed by RT-PCR at the time of hospitalization.

The cases in our study predominantly comprised males with a mean age of 60.8 years; they had more than one comorbidity with diabetes and hypertension being the most common. The histopathological findings in our study (Figure 7) were in line with the previous studies, with lung injury being the main finding in a majority (90.4%) of our patients [9,15,16]. In the lungs, the virus enters the alveolar epithelial and endothelial cells with the help of angiotensin-converting enzyme 2 (ACE2) receptors and triggers direct damage to the lung tissue [17]. A positive RT-PCR was seen in the lung tissue of 15 cases including the two cases that did not show any features of DAD in the present study, signifying that lungs are the most common organ to be affected by SARS-CoV-2. This is in concordance with the study by Wichmann et al. where 100% of the cases demonstrated RT-PCR positivity in the lung tissue [7]. The severity of the histopathological changes in lung tissue as well as the phase of DAD was neither affected by the duration of the disease nor the administration of mechanical ventilation or the SOFA score, which is in alignment with previous studies [18]. Polak et al. have described that though the epithelial, vascular, and fibrotic phases usually present at different times during the disease, they can occur simultaneously as well [19]. However, if fibrosis is present very early in the course of the disease, it is unlikely to be associated with COVID-19. In the present study, four cases showed evidence of fibrosis in the lung tissue in the early course of the disease, but no records of an already existing pulmonary fibrosis in these patients were available.

Liver injury in the form of moderately increased levels of AST and ALT was seen in around half of the cases in the present study, and the severity of histopathological findings correlated with the biochemical markers of AST and ALT, though the association was not statistically significant due to the low sample size. This is in concordance with the previous studies [20,21]. In the liver, though there is a higher expression of ACE2 enzyme in the ductular epithelial cells, no elevation in the bilirubin levels or any histologic change favoring ductular damage was evident in the present study; this aligns with a previous study by Fan et al. [21]. In the present study, 55% of cases were positive for SARS-CoV-2 in liver tissue on RT-PCR, which is comparable to the observations in previous studies; however, no evidence of hepatocyte injury was seen [21,22]. In contrast to previous studies, vascular thrombosis was not seen in the majority of the cases included in the present study even though the D-dimer levels were elevated in some of these cases [23,24]. A plausible reason for the same could be that all patients in the present study had received anticoagulants during the course of their treatment in the hospital.

In kidneys, the ACE2 enzyme is expressed on the apical brush border of proximal tubules and the podocytes of visceral epithelium, making them the key targets of SARS-CoV-2 [25]. ATN was the most prominent finding in the patients in the present study along with the histological changes related to diabetes and hypertension, similar to the observations in previous studies [26,27]. Almost half the cases (5/11 cases) with AKI clinically demonstrated moderate or severe ATN at autopsy, which is in contrast with the previous study by Santoriello et al. who found the severity of ATN to be mild as compared to the extent of rising in creatinine in 71% of their cases [13]. The occurrence of AKI in COVID-19 infection has been attributed to cytokine storm and the use of mechanical ventilation for acute respiratory distress syndrome (ARDS) in these cases [28]. However, in the present study, no significant association was established between the days of hospitalization or mechanical ventilation and the stage of AKI. In the majority of these cases, kidneys had already borne the brunt of preexisting chronic diseases, and the superimposed hypoxia induced by ARDS may have resulted in AKI, as suggested by Lax et al. [29]. There was no evidence of acute interstitial nephritis, acute glomerulonephritis, or collapsing glomerulopathy in any of the cases in the present study, which is in concordance with the study by Rivero et al. [30]. In 80% of cases, kidney tissue was found positive for RT-PCR, and hence the probability of direct viral effect on renal tissue cannot be ruled out.

Limitations

A small sample size, the lack of laboratory parameters, and immunohistochemical correlation for the presence of viral protein in the tissue samples were the major limitations of the study. Obtaining consent from the family members of the deceased was one of the biggest challenges faced and, as only the cases for which consent could be obtained were included in the study, the cohort of patients might not be truly representative of the broader population. The study was carried out in the midst of the first wave of the COVID-19 pandemic in India, with limited resources and funding for the study. The main limitation was the small number of cases studied as the consent for autopsy by the relatives was not forthcoming despite our best efforts.

Strengths

As the study was carried out in a tertiary care center, the treatment protocols for all patients in the cohort were similar and included the use of anticoagulants, steroids, and antibiotics. This uniformity of the treatment protocols was one of the major strengths of the study.

## Conclusions

The findings associated with lungs, liver, and kidneys in this autopsy study on 21 patients revealed that SARS-CoV-2 affects multiple systems, as evidenced by the presence of viral RNA in tissues in the majority of the cases; however, the severity of histopathological changes did not show any correlation with the clinical or biochemical parameters except in the liver. The presence of viral RNA in various organs also suggests that the virus spreads from the lungs through the bloodstream to infect the other organs. However, advanced studies are required to prove this hypothesis. The cause of death in the majority of the cases was lung injury, but the disease-associated mortality is multifactorial, and larger studies are required to determine the factors contributing to the severity of disease in some cases.
